# Quantitative Profiling of Oxylipin Reveals the Mechanism of Pien-Tze-Huang on Alcoholic Liver Disease

**DOI:** 10.1155/2021/9931542

**Published:** 2021-06-01

**Authors:** Ziye Zhu, Wenjun Zhou, Yang Yang, Kai Wang, Fenghua Li, Yanqi Dang

**Affiliations:** ^1^Institute of Digestive Diseases, Longhua Hospital, China-Canada Center of Research for Digestive Diseases (ccCRDD), Shanghai University of Traditional Chinese Medicine, Shanghai 200032, China; ^2^Experiment Center for Science and Technology, Shanghai University of Traditional Chinese Medicine, Shanghai 200032, China

## Abstract

Alcoholic liver disease (ALD) is a liver disease caused by long-term alcohol consumption. ROS-mediated oxidative stress is the leading cause of ALD. Pien-Tze-Huang (PZH), a traditional formula, is famous in China. This study was designed to evaluate the effects and explore the potential mechanisms of PZH in ALD. Forty mice were randomly divided into five groups: control group (normal diet + vehicle), model group (ethanol diet + vehicle), PZH-L group (ethanol diet + PZH (0.125 g/kg)), PZH-M group (ethanol diet + PZH (0.25 g/kg)), and PZH-H group (ethanol diet + PZH (0.5 g/kg)). The mice were sacrificed, and their liver and blood samples were preserved. Liver steatosis, triglyceride (TG), total cholesterol, serum alanine aminotransferase (ALT), and aspartate aminotransferase (AST) levels were assayed. Malondialdehyde (MDA), glutathione peroxidase (GSH-PX), and total superoxide dismutase were identified using commercial kits. Oxylipins were profiled, and the data were analyzed. The AMPK/ACC/CPT1A pathway was identified using real-time polymerase chain reaction and western blotting. The PZH-H intervention significantly alleviated hepatic steatosis and injury and reduced the levels of liver TG and serum ALT and AST. In addition, MDA levels were markedly reduced, and GSH-PX activity significantly increased after PZH-H intervention. Finally, PZH-H increased the levels of 17-HETE, 15-HEPE, 9-HOTrE, 13-HOTrE, and 5,6-dihydroxy-8Z,11Z,14Z,17Z-eicosatetraenoic acid, and reduced PGE2 levels. PZH-H intervention also promoted the phosphorylation of AMPK and ACC, and the expression of CPT1A. In conclusion, PZH reduced oxidative stress and alleviated hepatic steatosis and injury. The mechanism was correlated with the oxylipin metabolites/AMPK/ACC/CPT1A axis.

## 1. Introduction

Alcoholic liver disease (ALD) is a liver disease caused by long-term alcohol consumption. It is one of the most common types of chronic liver diseases globally and is currently one of the major chronic liver diseases in China [1–3]. Heavy drinkers (ethanol consumption of ≥40 g/day for men and ≥20 g/day for women over 5 years; or ethanol consumption of >80 g/day and binge drinking within 2 weeks) develop fatty liver, and about 20%–40% of them develop more severe ALD [1, 4]. According to the International Classification of Diseases (ICD-10), ALD is classified as alcoholic fatty liver, alcoholic hepatitis, alcoholic liver fibrosis, alcoholic cirrhosis, and alcoholic liver failure. Studies have found that the prevalence of ALD in China is 4.5%, which is similar to that in European and American countries, among which the prevalence of ALD in people aged 40–49 years is the highest, accounting for more than 10% [5]. At present, the effective ways to reduce or terminate alcoholic liver injury are nutritional support and abstinence from alcohol, but some patients still need to undergo combined treatment with drug intervention. The guidelines for the prevention and treatment in 2018 included glucocorticoid, metadoxine, S-adenosylmethionine, polyene phosphatidylcholine, glycyrrhizic acid preparation, and silymarin. Although these drugs show some improvement in patients with ALD, strict and extensive sample data supporting effective treatment and improvement of ALD are still lacking in clinical trials.

Alcohol consumption can induce the generation of reactive oxygen species (ROS), which cause oxidative stress. ROS can change the structure and function of the protein by binding to them and finally generate host antigens that can induce immune responses [6, 7]. ROS can also lead to lipid peroxidation and generate lipid peroxidation products, such as 4-hydroxynonenal (4-HNE) and malondialdehyde (MDA). Studies have found that these products can bind to DNA bases and induce apoptosis and autophagy in cells [8–10]. Therefore, ROS-mediated oxidative stress is the leading cause of ALD.

Oxylipins are produced by the oxidation of polyunsaturated fatty acids (PUFAs), including arachidonic acid (AA), linoleic acid (LA), eicosapentaenoic acid (EPA), and docosahexaenoic acid (DHA). Oxylipins play an important role in inflammatory response [11, 12], immune defense [13, 14], and oxidative stress [13, 15]. Studies have found that AA can inhibit inflammatory responses and oxidative stress to protect against brain injury [15]. LA attenuates acrolein-induced oxidative stress [16]. EPA can decrease ROS to attenuate oxidative stress [17–19]. In addition, DHA protects photoreceptors from oxidative stress [20]. These studies have shown that oxylipins can attenuate oxidative stress.

The traditional formula, Pien-Tze-Huang (PZH), is famous in China and is mainly composed of four traditional Chinese medicines, namely, *Panax notoginseng* (85%), Musk (3%), *Calculus bovis* (5%), and snake's gall bladder (7%). Studies have found that PZH has the functions of clearing away heat and detoxification, reducing swelling and relieving pain, anti-inflammatory effects, and liver protection [21–23]. Clinically, it is widely used in various inflammatory diseases, such as viral hepatitis, alcoholic hepatitis, and fatty liver [24]. Currently, the mechanism of PZH in ALD remains unclear. Therefore, in this study, we explored the protective effect of PZH on ALD and its potential mechanism.

## 2. Materials and Methods

### 2.1. Animals

C57BL/6 male mice (22–25 g) were obtained from Beijing Vital River Laboratory Animal Technology Co. Ltd, China. Mice were maintained under a specific pathogen-free environment (23–25°C, relative humidity: 40%–70%) with a 12-hour light/dark cycle.

### 2.2. Experimental Design and Administration of PZH

Mice were randomly divided into five groups: control group (normal diet + vehicle), model group (ethanol diet + vehicle), PZH-L group (ethanol diet + PZH (0.125 g/kg)), PZH-M group (ethanol diet + PZH (0.25 g/kg)), and PZH-H group (ethanol diet + PZH (0.5 g/kg)). Mice fed an ethanol diet were fed an ethanol Lieber-DeCarli diet (Trophic Animal Feed High-tech Co. Ltd, China) containing 5% (v/v) ethanol for 10 days. Mice with a normal diet were pair-fed with an equicaloric normal Lieber-DeCarli diet for 10 days. PZH was exclusively produced and certified by Zhangzhou Pien Tze Huang Pharmaceutical Co., Ltd., and the fingerprint spectrum was determined by HPLC-MS in a previous study [22]. PZH-treated mice were gavaged with PZH aqueous solution simultaneously as they were fed an ethanol diet for 10 days. On day 11, mice fed an ethanol diet were gavaged with a single dose of 31.5% (v/v) ethanol (20 *μ*L/g body weight), and mice fed a normal diet were gavaged with equicaloric 45% (w/v) maltose dextrin (20 *μ*L/g body weight). All mice were euthanized after 9 hours, and blood and liver samples were collected for further study.

### 2.3. Biochemical Analysis

The blood was centrifuged for 20 min at 4000 rpm at 4°C, and serum was collected for analysis. Liver tissue was homogenized in propanone/ethanol (1 : 1) and incubated overnight at 4°C. Homogenates were centrifuged for 10 min at 4000 rpm at 4°C, and the supernatant was collected for testing. Serum alanine aminotransferase (ALT) and aspartate aminotransferase (AST) levels, liver triglyceride (TG), and total cholesterol (TC) levels were measured using an automatic biochemistry analyzer (TBA-40FR, Toshiba, Japan).

### 2.4. Histological Analysis

The left lobe of the liver was cut in half. One half was fixed in 10% buffered formalin, and the other was frozen in liquid nitrogen. The fixed liver sections were dehydrated, embedded, and sectioned into 4 *μ*m sections for staining with hematoxylin and eosin (H&E). The frozen liver sections were embedded and sectioned into 10 *μ*m thick sections using a frozen microtome (Leica, Germany) for staining with Oil Red O, according to previous studies [25, 26].

### 2.5. Measurement of MDA, GSH-PX, and T-SOD Levels in the Liver

Liver tissue was homogenized and centrifuged for 10 min at 4000 rpm at 4°C, and the supernatant was collected. The levels of MDA, glutathione peroxidase (GSH-PX), and total superoxide dismutase (T-SOD) in liver tissue were measured using commercial kits (Nanjing Jiancheng Bioengineering Institute, Nanjing, China).

### 2.6. Sample Preparation and the Detection of Oxylipins Profile

Tissue (20 mg) was weighed and homogenized with 200 L of oxylipin extract (BB24, Next Advance, Inc., Averill Park, NY, USA) for 3 min. The mixture was centrifuged for 10 min at 5000 rpm at 4°C. The supernatant was concentrated, dried, and then redissolved in 100 L methanol/water (1 : 1, v/v). Seventy-one oxylipin metabolites were determined at the Metabo-Profile R & D Laboratory (Shanghai, China). Ultra-performance liquid chromatography-tandem mass spectrometry (UPLC-MS/MS) (ExionLC™ AD, QTRAP® 6500+) was used to detect them according to manufacturer's instructions.

### 2.7. Oxylipins Data Analysis

The raw data generated by UPLC-MS/MS were extracted, integrated, identified, and quantitatively analyzed for each metabolite using AB Sciex's Analyst software (V1.6.3, AB Sciex, Boston, MA, USA). Principal component analysis (PCA), partial least squares discriminant analysis (PLS-DA), and variable importance of projection (VIP) were performed to analyze the data. The formula used for calculating the *Z*-score was *Z*-scores = (x−mean)/SD. The Euclidean distance of Z-scores was clustered with the method in the heatmap. Kyoto Encyclopedia of Genes and Genomes (KEGG) pathways were enriched using Metaboanalyst 4.0.

### 2.8. Real-Time Polymerase Chain Reaction (RT-PCR)

Total RNA was isolated from liver tissue using RNAiso Reagent (Takara, Japan) and reverse-transcribed using PrimeScript™ RT Master Mix (Takara, Japan). RT-PCR was performed using TB Green® Premix Ex Taq™ according to the manufacturer's instructions. Target gene expression was analyzed using the 2^-ΔΔCt^ method by normalization to *β*-actin. The primers used are listed in Table 1.

### 2.9. Western Blot Analysis

Liver tissue was lysed and centrifuged for 10 min at 4000 rpm at 4°C, and the supernatant was collected and denatured. Denatured proteins were separated by SDS-PAGE and transferred to polyvinylidene fluoride membranes (Millipore, USA). The membranes were blocked using 5% skim milk and then incubated at 4°C overnight with the primary antibodies, including AMP-activated protein kinase alpha (AMPK*α*) (CST, USA), phospho-AMPK*α* (CST, USA), acetyl-CoA carboxylase (ACC) (CST, USA), phospho-ACC (CST, USA), carnitine palmitoyltransferase 1A (CPT1A) (Gene Tex), peroxisome proliferator-activated receptor alpha (PPAR*α*) (ABclonal, China), sterol regulatory element-binding transcription factor 1 (SREBP1) (Santa Cruz, USA), and HRP-conjugated *β*-actin (HUABIO, China). After the overnight incubation, the membranes were incubated with HRP-conjugated secondary antibody (Jackson Immuno). Blots were visualized with ECL luminescence reagent (Meilunbio, China) and quantified using ImageJ software.

### 2.10. Statistical Analysis

Data are presented as mean ± standard deviation ($\bar{x}\pm \text{s}$). Statistical significance was analyzed using one-way ANOVA in SPSS 24.0. Qualitative data among the three groups were assessed using one-way ANOVA, the Kruskal–Wallis test, or the chi-square test based on data distribution. Differences between the two groups were analyzed using Student's *t*-test. The values were considered statistically significant at *P* < 0.05.

## 3. Results

### 3.1. PZH-H Alleviated Hepatic Steatosis in ALD-Mice

**Figure 1:**
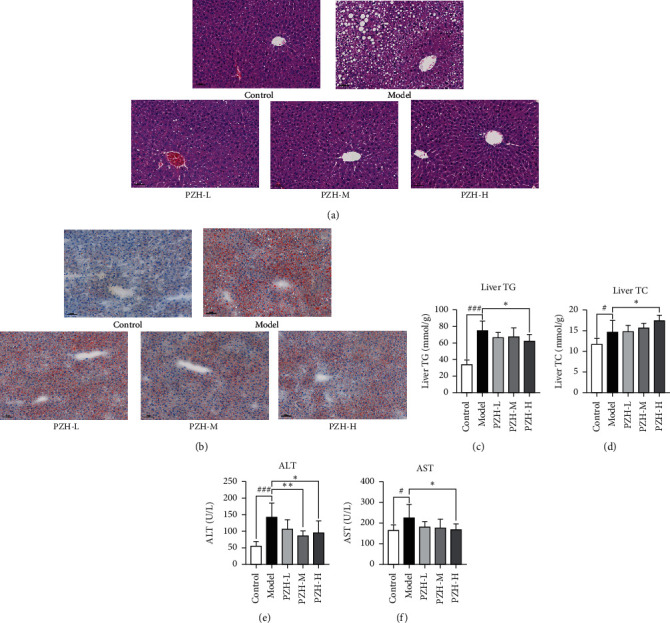
PZH-H alleviated hepatic steatosis in ALD-mice. (a) HE staining in liver sections was presented. (b) Oil Red O in liver sections was presented. (c) Liver TG and (d) TC were analyzed (*n* = 8); (e) Serum ALT and (f) AST were analyzed (*n* = 8). Data were presented as means ± SD. ^#^*P* < 0.05, ^##^*P* < 0.01, and ^###^*P* < 0.01 in the model group vs the control group; ^*∗*^*P* < 0.05 and ^*∗∗*^*P* < 0.01 in the PZH groups vs the model group.

Hepatic steatosis was significantly higher in the model group than in the control group, and the PZH-M and PZH-H interventions significantly improved hepatic steatosis (Figures 1(a) and 1(b)). In addition, liver TG and TC levels were markedly increased in the model group compared with those in the control group. The PZH-H intervention significantly reduced liver TG levels, but liver TC was increased in the PZH-H group compared with that in the model group (Figures 1(c) and 1(d)). Furthermore, serum ALT and AST levels were markedly increased in the model group compared with those in the control group. The PZH-H intervention markedly reduced ALT and AST levels, and the PZH-M intervention also markedly reduced ALT levels (Figures 1(e) and 1(f)). These results indicated that PZH-M and PZH-H alleviated hepatic steatosis and injury in ALD mice, and the effect of PZH-H was better than that of PZH-M.

### 3.2. Effect of PZH on MDA, GSH-PX, and SOD

**Figure 2:**
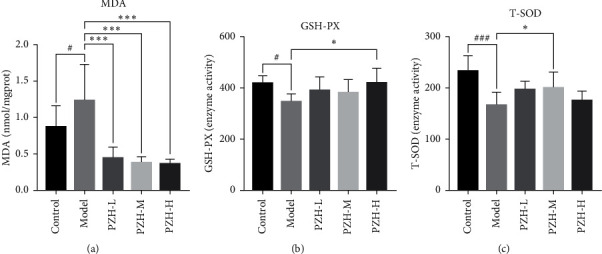
Effect of PZH on MDA, GSH-PX, and T-SOD. (a) The content of MDA, (b) the activity of GSH-PX, and (c) T-SOD was measured using kits (*n* = 8). Data were presented as means ± SD. ^#^*P* < 0.05 and ^###^*P* < 0.001 in the model group vs the control group; ^*∗*^*P* < 0.05 and ^*∗∗∗*^*P* < 0.001 in the PZH groups vs the model group.

Oxidative stress is one of the main pathogeneses of ALD; MDA, GSH-PX, and T-SOD are the primary biomarkers of oxidative stress. Thus, we tested the levels of MDA, GSH-PX, and T-SOD in the livers of ALD-mice. Compared with the control group, the level of MDA in the model group was significantly increased, while the activities of GSH-PX and T-SOD were significantly decreased. Compared with the model group, the level of MDA in the three PZH groups was significantly decreased (Figure 2(a)), and the activity of GSH-PX in the PZH-H group was significantly increased (Figure 2(b)). Although T-SOD activity in the PZH-H group was not different, its activity was significantly increased in the PZH-M group (Figure 2(c)). These results indicate that PZH-H could improve oxidative stress.

### 3.3. Analysis of Oxylipin Metabolites among Three Groups

**Figure 3:**
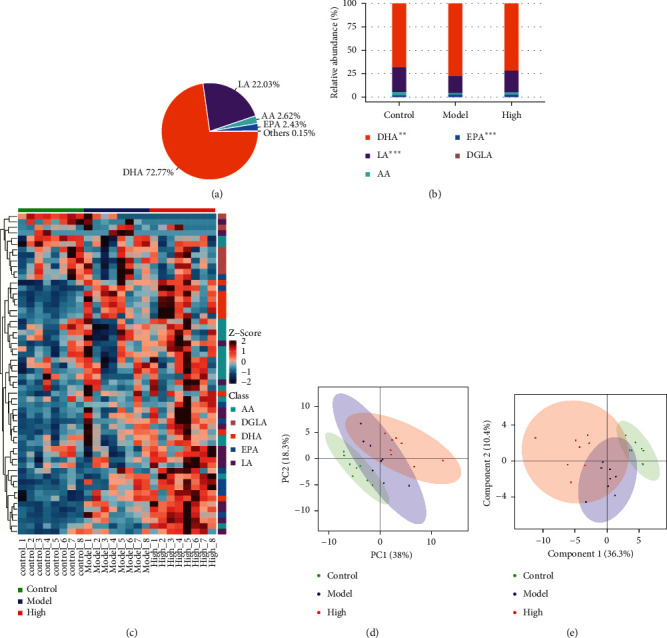
Analysis of oxylipin metabolites among three groups. (a) The proportion of identified metabolite classes in all groups was shown. (b) The relative abundance of each metabolite class in different groups was shown. (c) Fifty-eight oxylipin metabolites were detected in the liver tissue of mice. (d) PCA and (e) PLS-DA were analyzed and shown. High: PZH-H group.

Our results showed that PZH could alleviate hepatic steatosis, liver injury, and oxidative stress, and the effect of PZH-H was better than that of the PZH-L and PZH-M groups. Thus, we chose the PZH-H group to explore the mechanism of oxidative stress improvement. Previous studies have proved that oxylipins play an important role in oxidative stress [13, 15]. Therefore, the profiling of oxylipins was performed and analyzed. The results indicated that the proportion of DHA was the highest, followed by that of LA (Figure 3(a)). DHA, LA, and EPA levels were significantly different among the three groups (Figure 3(b)). Fifty-eight oxylipin metabolites were detected in the liver tissue of the mice (Figure 3(c), Supplementary file 1). The PCA and PLS-DA analyses yielded clear distinctions among the three groups (Figures 3(d) and 3(e)).

### 3.4. Effect of PZH on Different Oxylipin Metabolites

**Figure 4:**
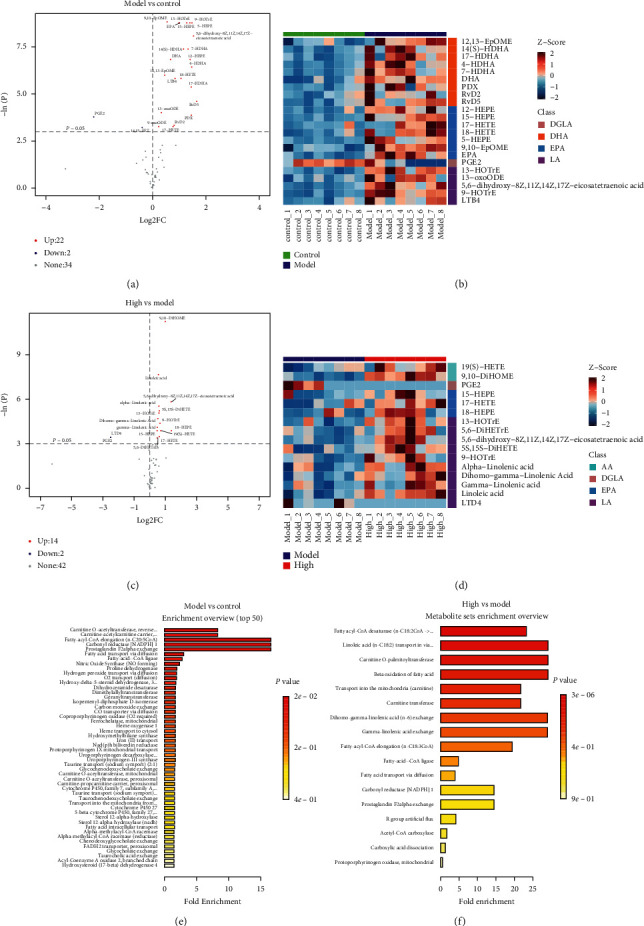
Effect of PZH on different oxylipin metabolites. (a) At *P* < 0.05, 24 different oxylipin-metabolites were obtained in the model group compared with the control group. (b) Combined with the value of VIP >1, 22 different oxylipin metabolites were obtained in the model group compared with the control group. (c) At *P* < 0.05, 16 different oxylipin-metabolites were obtained in the PZH-H group compared with the model group. (d) Combined with the value of VIP >1, 16 different oxylipin metabolites were obtained in the PZH-H group compared with the model group. The KEGG pathway was shown in (e) the model group compared with the control group and (f) the PZH-H group compared with the model group. High: PZH-H group.

**Figure 5:**
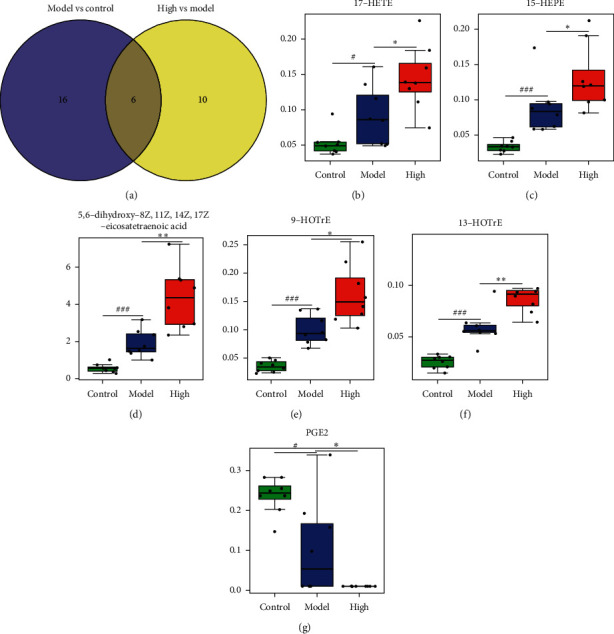
Effect of PZH on 6 overlapping oxylipin metabolites. (a) 6 overlapping oxylipin metabolites were showed among three groups. (b) 17-HETE, (c) 15-HEPE, (d) 5,6-dihydroxy-8Z,11Z,14Z,17Z-eicosatetraenoic acid, (e) 9-HOTrE, (f) 13-HOTrE, and (g) PGE2 levels were shown. ^#^*P* < 0.05 and ^###^*P* < 0.001 in the model group vs the control group; ^*∗*^*P* < 0.05 and ^*∗∗*^*P* < 0.01 in the PZH group vs the model group. High: PZH-H group.

First, using values of *P* < 0.05, the sets of 24 and 16 different oxylipin metabolites were obtained in the model group compared with the control group, and the PZH-H group compared with the model group (Figures 4(a) and 4(c)), respectively. Furthermore, combined with the value of VIP >1, only 22 and 16 different oxylipin metabolites were obtained in the model group compared with the control group, and the PZH-H group compared with the model group (Figures 4(b) and 4(d), Supplementary file 2), respectively. KEGG pathway analysis revealed that the main pathways included carnitine O-acetyltransferase, carnitine-acetylcarnitine carrier, fatty acyl-CoA desaturase, linoleic acid (n-C18 : 2) transport via diffusion, carnitine O-palmitoyltransferase, and beta-oxidation of fatty acids (Figures 4(e) and 4(f)). We further identified six overlapping oxylipin metabolites among the three groups using a Venn diagram (Figure 5(a)), including two EPAs (17-HETE and 15-HEPE), three LAs (9-HOTrE, 13-HOTrE, 5,6-dihydroxy-8Z, 11Z, 14Z, 17Z-eicosatetraenoic acid), and one DGLA (PGE2). The levels of 17-HETE, 15-HEPE, 5,6-dihydroxy-8Z, 11Z, 14Z, 17Z-eicosatetraenoic acid, 9-HOTrE, and 13-HOTrE were markedly increased, and PGE2 levels were significantly reduced in the model group compared with the control group. PZH-H intervention further increased the levels of 17-HETE, 15-HEPE, 9-HOTrE, 13-HOTrE, and 5,6-dihydroxy-8Z, 11Z, 14Z, 17Z-eicosatetraenoic acid, and reduced PGE2 levels (Figures 5(b)–5(g), Table 2).

### 3.5. Oxylipin Metabolites Were Negatively Correlated with MDA

**Figure 6:**
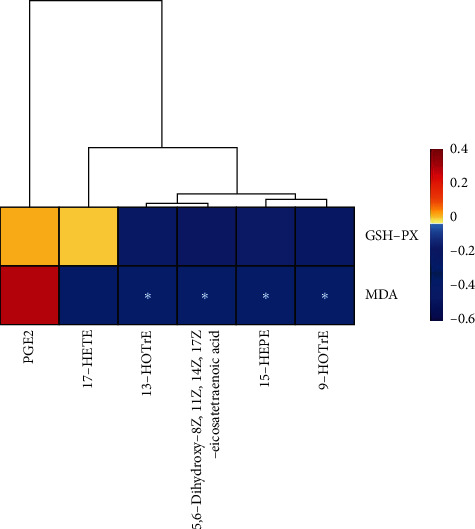
The correlation of oxylipin-metabolites, MDA, and GSH-PX was analyzed by the Spearman correlation analysis.

PZH-H reduced MDA levels and promoted the activity of GSH-PX (Figure 2). Profiling of oxylipins indicated that PZH-H could also increase the levels of 17-HETE, 15-HEPE, 5,6-dihydroxy-8Z, 11Z, 14Z, 17Z-eicosatetraenoic acid, 9-HOTrE, 13-HOTrE, and reduced PGE2 levels (Figure 4). Therefore, Spearman correlation analysis was performed to evaluate the correlation of oxylipin metabolites, MDA, and GSH-PX. The results showed that 15-HEPE, 5,6-dihydroxy-8Z, 11Z, 14Z, 17Z-eicosatetraenoic acid, 9-HOTrE, and 13-HOTrE were negatively correlated with MDA, and there was no correlation among the other indices (Figure 6).

### 3.6. Effect of PZH on AMPK-ACC-CPT1A Pathway

**Figure 7:**
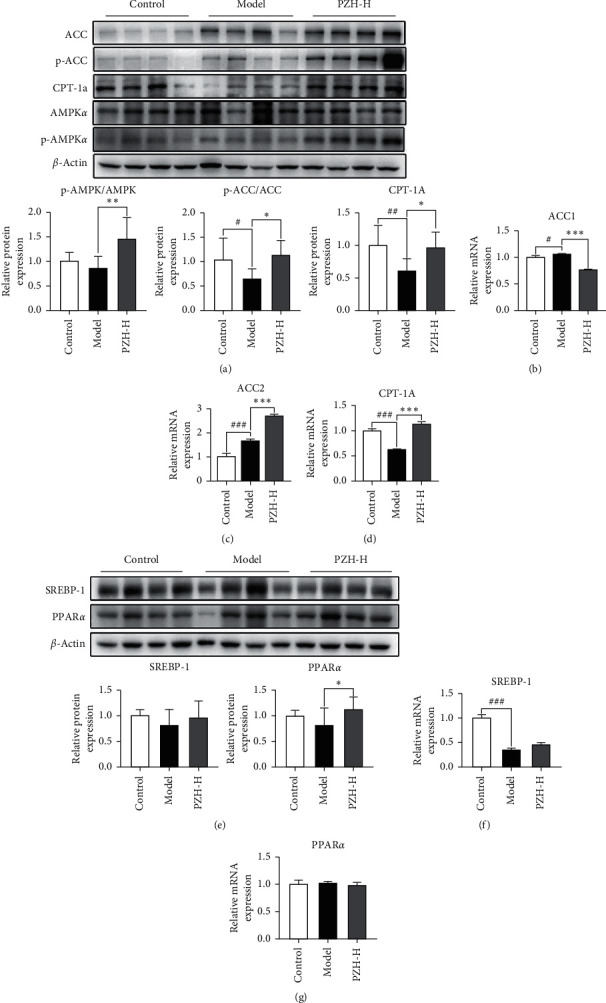
Effect of PZH on AMPK-ACC-CPT1A pathway. (a) The levels of p-AMPK, AMPK, p-ACC, ACC, and CPT-1A were shown (*n* = 8). The mRNA expression of (b) ACC1, (c) ACC2, and (d) CPT-1A was detected by RT-PCR (*n* = 8). (e) The protein expression of SREBP-1 and PPAR*α* was shown (*n* = 8). (f) SREBP-1 mRNA expression and (g) PPAR*α* mRNA expression were detected (*n* = 8). Data were presented as means ± SD. ^#^*P* < 0.05, ^##^*P* < 0.01, and ^###^*P* < 0.001 in the model group vs the control group; ^*∗*^*P* < 0.05, ^*∗∗*^*P* < 0.01, and ^*∗∗∗*^*P* < 0.001 in the PZH-H group vs the model group.

Previous studies have indicated that oxylipin metabolites can regulate the AMPK signaling pathway [27–30]. The results showed that although the phosphorylation of AMPK was not significantly different in the model group compared with the control group, PZH-H intervention markedly promoted the phosphorylation of AMPK (Figure 7(a)). In addition, compared with the control group, the phosphorylation of ACC was significantly reduced in the model group, and PZH-H intervention markedly promoted the phosphorylation of ACC. However, the mRNA levels of ACC1 and ACC2 in the model group were significantly increased, the mRNA levels of ACC1 were significantly decreased, and ACC2 levels were significantly increased after PZH-H intervention (Figures 7(a)–7(c)). Compared with the control group, the mRNA and protein levels of CPT1A were significantly decreased in the model group, and PZH-H intervention significantly promoted CPT1A expression (Figures 7(a) and 7(d)). Although the phosphorylation of PPAR*α* was not significantly different in the model group compared with the control group, the PZH-H intervention markedly promoted the phosphorylation of PPAR*α*. Moreover, the expression of SREBP1 was not significantly different among the three groups (Figures 7(e)–7(g)).

## 4. Discussion

In the present study, we showed that PZH could improve hepatic steatosis and injury in ALD mice, consistent with previous studies. In addition, PZH reduced the level of MDA and increased the activity of GSH-PX, ultimately ameliorating oxidative stress, which is consistent with a previous study [31]. Through the analysis of oxylipin profiling, we found that PZH promoted the levels of 17-HETE, 15-HEPE, 5,6-dihydroxy-8Z, 11Z, 14Z, 17Z-eicosatetraenoic acid, 9-HOTrE, and 13-HOTrE, and reduced PGE2 levels, further activating the AMPK pathway. Previous studies have also shown that PZH can reduce tumor necrosis factor-alpha and interleukin-1*β* secretion to ameliorate hepatic inflammation [31] and can ameliorate hepatic injury by inhibiting the PERK/eIF2*α* pathway [22]. In addition, PZH could also ameliorate hepatic fibrosis by suppressing the NF-kappa B pathway [21]. Panax notoginseng saponins and polysaccharides have hepatoprotective effects against alcoholic liver damage [32, 33], but the mechanism is not precise. Our study is the first to demonstrate that PZH regulates the oxylipin-metabolites/AMPK pathway to reduce oxidative stress and improve hepatic steatosis and injury in ALD-mice. This study fully confirmed the mechanism of action of PZH on ALD through the quantitative profiling of oxylipin.

Oxidative stress is one of the main pathogeneses of ALD. As a primary biomarker of oxidative stress, MDA was markedly increased in ALD and promoted the generation of etheno-DNA adducts to induce damage to the liver [9, 10]. In the present study, MDA levels were also markedly increased in the ALD mice, and PZH intervention significantly reduced MDA levels. In addition, the activity of GSH-PX, an antioxidant enzyme, was significantly reduced in ALD-mice, consistent with a previous study [34], and PZH increased the activity. These results indicated that PZH could attenuate oxidative stress.

Oxylipins play an essential role in oxidative stress [13, 15]. Studies have shown that EPA, as an oxylipin, can decrease oxidative stress and placental lipid deposition in a sirtuin-1-independent manner [17]. The EPA could also decrease oxidative stress and attenuate cardiomyoblast apoptosis by activating autophagy [35]. EPA supplementation attenuates HFD-induced oxidative stress and steatosis [36]. In this study, PZH promoted the levels of EPA (17-HETE, 15-HEPE). Furthermore, LA is also an oxylipin, and studies have shown that LA can attenuate acrolein-induced oxidative stress [16]. Clinical trials have confirmed that LA can improve oxidative stress in obese patients with non-alcoholic fatty liver disease [37]. In our study, PZH increased LA levels, including 9-HOTrE, 13-HOTrE, and 5,6-dihydroxy-8Z, 11Z, 14Z, 17Z-eicosatetraenoic acid. Studies have also shown that high DGLA levels could be a biomarker for hepatic steatosis [38]. PGE2 can promote hepatic fibrosis and lead to insulin resistance [39]. Green tea extract attenuated PGE2 accumulation and protected against liver injury in an HFD-feeding NASH model [40], and meloxicam reduced PGE2 level and modulated oxidative stress to protect against liver injury [41]. Moreover, *Panax notoginseng* saponins attenuated gastric injury by modulating the metabolism of PGE2 [42]. In the present study, we found that PZH reduced PGE2 levels. In addition, our results further proved that 15-HEPE, 9-HOTrE, 13-HOTrE, and 5,6-dihydroxy-8Z, 11Z, 14Z, 17Z-eicosatetraenoic acid were negatively correlated with MDA. Further analysis showed that the upregulated metabolites (15-HEPE, 5,6-dihydroxy-8Z, 11Z, 14Z, 17Z-eicosatetraenoic acid, 9-HOTrE, and 13-HOTrE) were negative correlated with T-SOD, and downregulated PGE2 were positively correlated with T-SOD (Supplementary Figure 1), indicating that PZH could reduce oxidative stress through non-SOD dependent approach. Finally, the results showed that PZH may improve oxidative stress by oxylipin metabolites, including EPA, LA, and PGE2. On the basis of a previous study [42], we speculated that *Panax notoginseng* of PZH may mainly enhance oxylipin metabolites.

The AMPK-ACC-CPT1A pathway is involved in the regulation of oxidative stress and ALD [43–47]. Previous studies have proved that EPA could improve palmitate-induced endothelial dysfunction and lipotoxicity by activating AMPK pathway [28, 48]. In addition, LA can reduce hepatic lipid metabolism [29, 30], insulin resistance, and adiposity [49, 50] via the AMPK pathway. PGE2 can negatively regulate the AMPK pathway [51, 52]. In this study, we found that PZH increased the levels of EPA and LA and reduced PGE2 levels. In addition, PZH can activate AMPK and promote the phosphorylation of ACC, which reduces the ACC activity [53] and finally promotes the expression of CPT1A. Although studies have shown that PPAR*α* and SREBP-1 could improve oxidative stress [54–56], PZH did not regulate their expression in our study, indicating that PZH did not play a regulatory role in ALD through PPAR*α* and SREBP-1. However, in this study, we found that the levels of 9-HOTrE, 13-HOTrE, 5,6-dihydroxy-8Z, 11Z, 14Z, 17Z-eicosatetraenoic acid, 17-HETE, and 15-HEPE were increased in the liver of ALD-mice, and PZH further increased their levels. PGE2 levels were decreased in the liver of ALD-mice, and PZH further decreased this level. We speculated that oxylipin metabolites may need to reach certain concentrations in vivo and activate the AMPK/ACC/CPT1A pathway to protect against hepatic injury. However, further studies are required to elucidate the underlying mechanism. Although our results showed that PZH could regulate oxylipin metabolites and speculated that Panax notoginseng may mainly enhance oxylipin metabolites, further studies are also needed.

## 5. Conclusions

**Figure 8:**
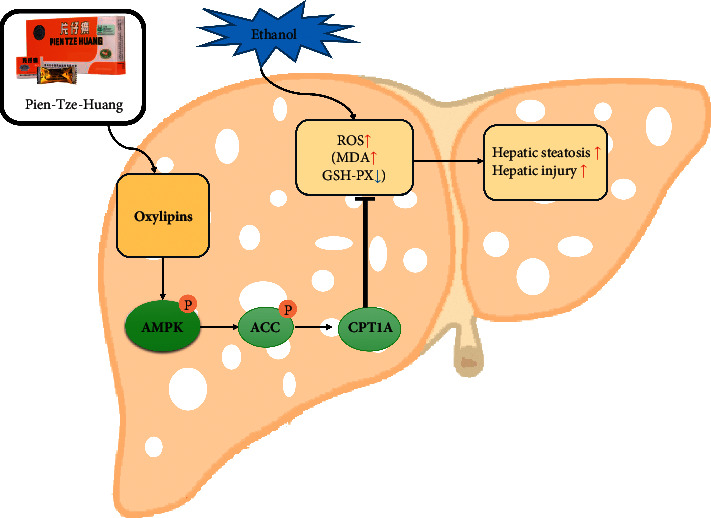
Summary of the study. Traditional Chinese formulae PZH increased the levels of oxylipin metabolites, activated AMPK-ACC-CPT1A pathway, and finally alleviated oxidative stress and hepatic steatosis.

In summary, PZH reduced oxidative stress and alleviated hepatic steatosis and injury. The mechanism was correlated with the oxylipin metabolites/AMPK/ACC/CPT1A pathway (Figure 8).

## Figures and Tables

**Table 1:** The sequence of primers in the study.

Primer name	Forward primer sequence (5′⟶3′)	Reverse primer sequence (5′⟶3′)
*β*-Actin	GGCTGTATTCCCCTCCATCG	CCAGTTGGTAACAATGCCATGT
ACC1	GATGAACCATCTCCGTTGGC	GACCCAATTATGAATCGGGAGTG
ACC2	CCTTTGGCAACAAGCAAGGTA	AGTCGTACACATAGGTGGTCC
CPT1A	CTCCGCCTGAGCCATGAAG	CACCAGTGATGATGCCATTCT
PPAR*α*	AGAGCCCCATCTGTCCTCTC	ACTGGTAGTCTGCAAAACCAAA
SREBP-1	GCAGCCACCATCTAGCCTG	CAGCAGTGAGTCTGCCTTGAT

**Table 2:** Six overlapping oxylipin metabolites among three groups.

Class	Metabolite	Model vs. control		PZH-H vs. model			
		Fold change	*P* value	VIP	Fold change	*P* value	VIP
EPA	17-HETE	1.735887097	0.037917638	1.059880581	1.574488	0.030620	1.438892
EPA	15-HEPE	2.479289941	0.0001554	1.470827013	1.431981	0.020668	1.013007
LA	5,6-Dihydroxy-8Z, 11Z, 14Z, 17Z-eicosatetraenoic acid	2.960944596	0.0003108	1.678973419	2.665644	0.002953	1.914047
LA	9-HOTrE	2.685344828	0.0001554	1.810016764	1.625732	0.012600	1.562081
LA	13-HOTrE	2.027124774	0.0001554	1.770831487	1.554011	0.006302	1.812153
DGLA	PGE2	0.220679012	0.022819743	1.13067223	0.181818	0.032474	1.211364

## Data Availability

The data used to support the findings of this study are available from the corresponding author upon request.

## Supplementary Materials

Supplementary Materials
*Supplementary file 1*. The levels of oxylipin-metabolites are shown among three groups. *Supplementary file 2*. 22 and 16 different oxylipin-metabolites are shown in the model group compared with the control group, and the PZH-H group compared with the model group, respectively*. Supplementary Figure 1*. The correlation of oxylipin-metabolites and T-SOD was analyzed by the Spearman correlation analysis.
