# Supplementary material for: Quantitative Profiling of Oxylipin Reveals the Mechanism of Pien-Tze-Huang on Alcoholic Liver Disease
Source: Evid Based Complement Alternat Med. 2021 Jun 1;2021:9931542. doi: 10.1155/2021/9931542 (PMC8187045; doi:10.1155/2021/9931542)
Supplement: Supplementary Materials — Supplementary file 1. The levels of oxylipin-metabolites are shown among three groups. Supplementary file 2. 22 and 16 different oxylipin-metabolites are shown in the model group compared with the control group, and the PZH-H group compared with the model group, respectively. Supplementary Figure 1. The correlation of oxylipin-metabolites and T-SOD was analyzed by the Spearman correlation analysis. [file 9931542.f1.zip › 9931542.f1/Supplementary file 1.pdf]

Supplementary file 1

The levels of oxylipin-metabolites were showed among three groups.

Metabolite	Control.Mean	Control.SD	Model.Mean	Model.SD	PZH-H.Mean	PZH-H.SD
20-HETE	0.0111975	0.004276359	0.01130625	0.00532958	0.016875	0.005806093
5-HETE	1.8575	0.403334317	2.01375	0.63053124	2.28875	0.540223168
19(S)-HETE	0.0483625	0.014078955	0.0802125	0.03699117	0.135725	0.047021933
18-HETE	0.051075	0.017346037	0.1089625	0.03791697	0.147925	0.035688964
17-HETE	0.05275	0.017885828	0.0922125	0.04214171	0.1451875	0.045847525
16-HETE	0.0377125	0.011817715	0.031825	0.01382728	0.043875	0.01070257
15-HETE	1.9925	0.424491292	2.065	0.85306171	2.325	0.640646994
12-HETE	2.20375	0.635181358	2.18875	0.73884925	2.95375	1.435279341
11(S)-HETE	1.1965	0.313167231	1.314875	0.42410761	1.4225	0.27525313
9-HETE	1.004375	0.322411422	1.270125	0.68804702	1.291375	0.309013609
14,15-EET	0.2685	0.058331565	0.219	0.06924284	0.2105	0.039478747
11,12-EET	0.18345	0.042480483	0.1673125	0.04523979	0.172375	0.034969119
8,9-EET	0.1110375	0.023826272	0.12485	0.03343664	0.1329	0.032960062
5,6-EET	7.41625	1.819622704	6.42875	2.91938564	8.81375	3.278762561
9-oxoODE	2.215	0.704617424	2.845	0.87948037	3.62875	1.40619484
13-oxoODE	1.178125	0.119281704	1.505	0.28334734	1.8525	0.536809357
18-HEPE	0.1081625	0.046026916	0.1573125	0.09482374	0.2705	0.104543907
15-HEPE	0.0338	0.007576279	0.0891	0.03772407	0.1308875	0.046319617
12-HEPE	0.0955375	0.047450514	0.256875	0.10906543	0.579	0.457724808
5-HEPE	0.0520375	0.013509567	0.1596375	0.07656332	0.233875	0.069107447
EPA	25.4125	5.336782739	52.1625	13.0992843	63.7375	19.19389319
AA	35.7875	31.14571456	22.60875	16.5776523	35.05	22.12496973
DHA	998.625	240.1897687	1530	286.256628	1628.75	258.3152835
ɔ-gamma-Linolen	174.5	36.04362436	149.8125	36.0851547	221.5	54.62861626
Linoleic acid	140.4375	27.83810018	118.95	27.2308648	180.25	26.87404483
lpha-Linolenic ac	42.5875	12.16534158	55.875	17.5830072	86.775	18.29143047
mma-Linolenic ac	39.85	7.884704904	38.225	9.28620328	55.05	14.3438389
4-HDHA	8.28625	3.482441416	26.375	11.6746061	28.4375	10.27867939
7-HDHA	4.2575	1.856492469	11.3725	4.97941118	11.71125	4.922483222
14(S)-HDHA	13.96	4.821413545	34.15	12.6743836	38.4125	18.36576298
17-HDHA	5.0025	2.677017263	15.5375	7.22512036	20.13875	16.41482991
RvD2	0.000991725	0.000667053	0.00230625	0.00159601	0.00226475	0.001136276
PGD1	0.18025	0.043506157	0.191375	0.07147215	0.180125	0.058403614
6-Keto-PGF1a	0.878	0.32176966	0.8965	0.5109171	0.578	0.232293534
PGF2a	0.85975	0.31525126	0.68075	0.24022177	0.6935	0.249349439
PGF1a	0.191375	0.069691027	0.183625	0.03994975	0.200875	0.05666805
PGJ2	0.89075	0.333553915	0.93575	0.60370351	0.860625	0.201633286
LTB4	0.0130575	0.005064797	0.0269	0.01344385	0.029625	0.011343689
TXB2	0.344625	0.118191053	0.313625	0.16544048	0.255125	0.118127579
Lipoxin A4	0.06395	0.012370703	0.08965	0.04284694	0.1240375	0.045249718
9,10-DiHOME	1.042	0.230840081	1.06675	0.23681382	2.1925	0.378295047
16(17)-EpDPE	0.08045	0.033007921	0.105775	0.04588538	0.136375	0.047875337
5-HETrE	0.0987625	0.079397984	0.151375	0.1137954	0.1714	0.118639875
,11Z,14Z,17Z-eic	0.559625	0.23229657	1.895	0.72377976	4.33875	1.656626844

5S,15S-DiHETE	0.040625	0.015518261	0.0470375	0.01569558	0.0743625	0.01748795
5-oxoETE	1.2495	0.24942448	1.624	1.00504342	1.65875	0.3632566
15-oxoETE	0.7785	0.135744508	0.865375	0.33789683	0.923375	0.234715595
5,6-DiHETrE	0.1006	0.037737287	0.1260625	0.02867109	0.18075	0.056380721
9,10-EpOME	0.304625	0.055597887	0.452875	0.05928488	0.537	0.092939304
12,13-EpOME	0.341125	0.061182251	0.476875	0.08232937	0.5355	0.072111026
9-HOTrE	0.0359	0.010141992	0.1002625	0.02593519	0.163	0.052704025
13-HOTrE	0.025825	0.006299603	0.0593625	0.01614222	0.09225	0.023328523
RvD5	0.0013489	0.001088776	0.00433875	0.00242011	0.00551375	0.003541008
PDX	0.0796625	0.043784535	0.237	0.15003904	0.2745	0.25821807
PGE1	0.002751925	0.002396493	0.00710053	0.00898259	0.003590125	0.005760891
PGE2	0.2365	0.044612618	0.1031875	0.12038879	0.00975	0
LTD4	0.0034875	0.003062067	0.00201438	0.00309215	7.25E-05	0
LXA5	0.03123	0.043619528	0.0594	0.07449123	0.00824	0
