# Supplementary material for: Quantitative Profiling of Oxylipin Reveals the Mechanism of Pien-Tze-Huang on Alcoholic Liver Disease
Source: Evid Based Complement Alternat Med. 2021 Jun 1;2021:9931542. doi: 10.1155/2021/9931542 (PMC8187045; doi:10.1155/2021/9931542)
Supplement: Supplementary Materials — Supplementary file 1. The levels of oxylipin-metabolites are shown among three groups. Supplementary file 2. 22 and 16 different oxylipin-metabolites are shown in the model group compared with the control group, and the PZH-H group compared with the model group, respectively. Supplementary Figure 1. The correlation of oxylipin-metabolites and T-SOD was analyzed by the Spearman correlation analysis. [file 9931542.f1.zip › 9931542.f1/Supplementary file 2.pdf]

Supplementary file 2

22 and 16 different oxylipin-metabolites were showed in the model group compared with the control group, and the PZH-H group compared with the model group, respectively.

Model vs Control

Class	Metabolite	P	FDR	FC	log2FC	VIP
EPA	18-HETE	0.002943	0.011417	2.133382	1.093143	1.427688847
EPA	17-HETE	0.037918	0.095618	1.735887	0.795673	1.059880581
LA	13-oxoODE	0.018044	0.058142	1.281513	0.357848	1.211929252
EPA	15-HEPE	0.000155	0.001502	2.47929	1.309927	1.470827013
EPA	12-HEPE	0.001088	0.005736	2.704819	1.435532	1.513395351
EPA	5-HEPE	0.000155	0.001502	2.83825	1.505002	1.511629163
EPA	EPA	0.000155	0.001502	2.039604	1.028289	1.65209901
DHA	DHA	0.001088	0.005736	1.623596	0.699192	1.439910345
DHA	4-HDHA	0.001616	0.007812	2.82273	1.497091	1.675761154
DHA	7-HDHA	0.000622	0.004006	2.567308	1.360256	1.545721899
DHA	14(S)-HDHA	0.000622	0.004006	2.281787	1.190164	1.632493436
DHA	17-HDHA	0.004662	0.0169	2.782294	1.476275	1.485835109
DHA	RvD2	0.035556	0.095618	1.787365	0.837834	1.100355698
LA	LTB4	0.002953	0.011417	1.827309	0.869721	1.225782805
LA	Z,11Z,14Z,17Z-eico	0.000311	0.002575	2.960945	1.566057	1.678973419
EPA	9,10-EpOME	0.000147	0.001502	1.486664	0.572079	1.55136128
DHA	12,13-EpOME	0.002482	0.011075	1.397948	0.483311	1.312873497
LA	9-HOTrE	0.000155	0.001502	2.685345	1.425107	1.810016764
LA	13-HOTrE	0.000155	0.001502	2.027125	1.019435	1.770831487
DHA	RvD5	0.010051	0.034291	3.21651	1.685496	1.277079374
DHA	PDX	0.020769	0.063399	2.795485	1.483098	1.37901668
DGLA	PGE2	0.02282	0.066177	0.220679	-2.17998	1.13067223

PZH-H vs Model

Class	Metabolite	P	FDR	FC	log2FC	VIP
AA	19(S)-HETE	0.020727	0.100180	1.692068	0.758788	1.241024
EPA	17-HETE	0.030620	0.122095	1.574488	0.654883	1.438892
EPA	18-HEPE	0.020668	0.100180	2.701357	1.433685	1.096781
EPA	15-HEPE	0.020668	0.100180	1.431981	0.518012	1.013007
LA	no-gamma-Linoleni	0.009135	0.075686	1.478515	0.564149	1.546600
LA	Linoleic acid	0.000470	0.013627	1.515343	0.599644	1.808107
LA	alpha-Linolenic acid	0.003954	0.057336	1.553020	0.635077	1.793215
LA	amma-Linolenic aci	0.016507	0.100180	1.440157	0.526226	1.546347
AA	9,10-DiHOME	0.000013	0.000772	2.055308	1.039355	1.887135
LA	Z,11Z,14Z,17Z-eico	0.002953	0.057084	2.665644	1.414484	1.914047
LA	5S,15S-DiHETE	0.005451	0.060920	1.580919	0.660764	1.289418
LA	5,6-DiHETrE	0.033681	0.122095	1.433813	0.519856	1.507663
LA	9-HOTrE	0.012600	0.091352	1.625732	0.701090	1.562081
LA	13-HOTrE	0.006302	0.060920	1.554011	0.635997	1.812153

DGLA	PGE2	0.032474	0.122095	0.181818	-2.459432	1.211364
LA	LTD4	0.032474	0.122095	0.181818	-2.459432	1.153951
