# Supplementary figures and images for: Quantitative Profiling of Oxylipin Reveals the Mechanism of Pien-Tze-Huang on Alcoholic Liver Disease
Source: Evid Based Complement Alternat Med. 2021 Jun 1;2021:9931542. doi: 10.1155/2021/9931542 (PMC8187045; doi:10.1155/2021/9931542)

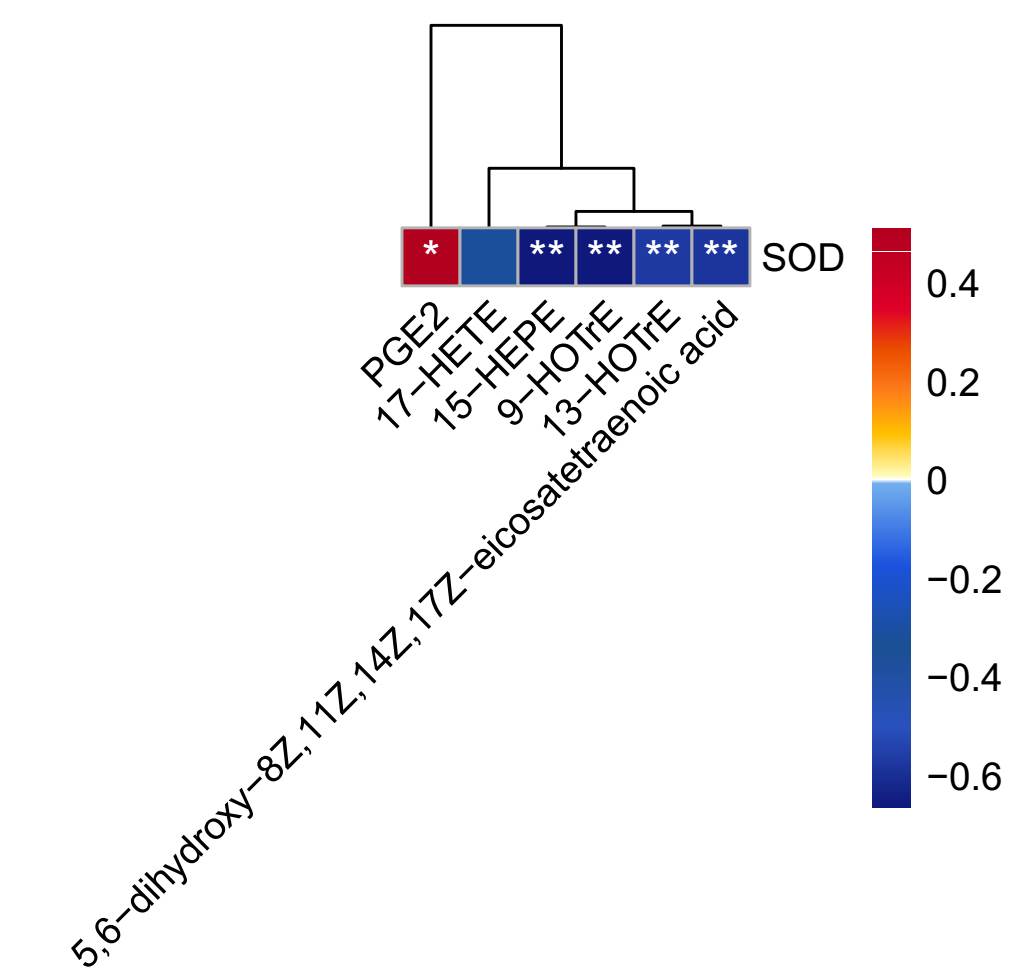

Supplement: Supplementary Materials — Supplementary file 1. The levels of oxylipin-metabolites are shown among three groups. Supplementary file 2. 22 and 16 different oxylipin-metabolites are shown in the model group compared with the control group, and the PZH-H group compared with the model group, respectively. Supplementary Figure 1. The correlation of oxylipin-metabolites and T-SOD was analyzed by the Spearman correlation analysis. [file 9931542.f1.zip › 9931542.f1/supplementary figure 1.jpg]
